# Regional Variation in Travel-related Illness acquired in Africa, March 1997–May 2011

**DOI:** 10.3201/eid2004.131128

**Published:** 2014-04

**Authors:** Marc Mendelson, Pauline V. Han, Peter Vincent, Frank von Sonnenburg, Jakob P. Cramer, Louis Loutan, Kevin C. Kain, Philippe Parola, Stefan Hagmann, Effrossyni Gkrania-Klotsas, Mark Sotir, Patricia Schlagenhauf, for the GeoSentinel Surveillance Network

**Affiliations:** University of Cape Town Groote Schuur Hospital, Cape Town, South Africa (M. Mendelson);; Centers for Disease Control and Prevention, Atlanta, Georgia, USA (P.V. Han, M. Sotir);; Tokai Medicross Travel Clinic, Cape Town (P. Vincent);; University of Munich, Munich, Germany (F. von Sonnenburg);; University Medical Center, Hamburg-Eppendorf, Germany (J.P. Cramer);; University of Geneva, Geneva, Switzerland (L. Loutan);; University of Toronto, Toronto, Ontario, Canada (K.C. Kain);; Assitance Publique Hôpitaux de Marseille–North University Hospital, Marseille, France (P. Parola);; Yeshiva University Bronx-Lebanon Hospital Center, Bronx, New York, USA (S. Hagmann);; Cambridge University Hospitals National Health Service Trust, Cambridge, UK (E. Gkrania-Klotsas);; University of Zurich Centre for Travel Medicine, Zurich, Switzerland (P. Schlagenhauf)

**Keywords:** travel, Africa, malaria, diarrhea, vaccine, HIV, viruses, parasites, helminth, bacteria, tuberculosis and other mycobacteria, endemic, vector, plasmodium, falciparum, vivax, ovale, malariae, schistosomiasis, strongyloidiasis, dengue, podcast, enteric, respiratory, rabies, zoonoses, vector-borne infections

## Abstract

To understand geographic variation in travel-related illness acquired in distinct African regions, we used the GeoSentinel Surveillance Network database to analyze records for 16,893 ill travelers returning from Africa over a 14-year period. Travelers to northern Africa most commonly reported gastrointestinal illnesses and dog bites. Febrile illnesses were more common in travelers returning from sub-Saharan countries. Eleven travelers died, 9 of malaria; these deaths occurred mainly among male business travelers to sub-Saharan Africa. The profile of illness varied substantially by region: malaria predominated in travelers returning from Central and Western Africa; schistosomiasis, strongyloidiasis, and dengue from Eastern and Western Africa; and loaisis from Central Africa. There were few reports of vaccine-preventable infections, HIV infection, and tuberculosis. Geographic profiling of illness acquired during travel to Africa guides targeted pretravel advice, expedites diagnosis in ill returning travelers, and may influence destination choices in tourism.

Africa is a popular tourist destination and a focus for international aid work, research, and business travel. In 2011, compared with 2010, international arrivals on the African continent remained relatively stable at ≈50 million, although the number of travelers to northern Africa decreased by 1.7 million (9%) and travel to sub-Saharan Africa increased by 1.3 million (4%) travelers (*1*). Identification of the types and relative frequencies of illnesses acquired by travelers to Africa would enable targeted prevention strategies before and during travel, as well as diagnosis and management of illness in returnees.

Previously described as “the Dark Continent,” referring to poor knowledge of its interior, Africa’s diverse geography, ecosystems, and climate are now well defined. However, detailed understanding is lacking about the variety of illnesses experienced by travelers who visit different parts of this diverse continent. Prior studies have focused on travel-related illnesses acquired only within sub-Saharan Africa (*2*) or have concentrated on a single infection (*3*). The perception of the public and health care practitioners is that the risk of acquiring many travel-related illnesses, including malaria, is uniform throughout the continent. This misconception was particularly evident to practitioners of travel health in South Africa before the 2010 Fédération Internationale de Football Association World Cup in South Africa, an annual event that attracts hundreds of thousands of persons to the hosting country. This erroneous concept and a need for evidence-based travel advice prompted a GeoSentinel study that demonstrated marked variation in morbidity rates for malaria, African tick bite fever, and other travel-related illnesses in persons returning from South Africa compared with persons returning from neighboring countries or other countries in sub-Saharan Africa (*4*). The objective of the current analysis is to study regional variation in travel-acquired and emerging infections across Africa. Reaching this objective will better inform pretravel clinical consultations, which will help travelers recognize illnesses in the travel destination, take preventive measures, and seek treatment; and will focus differential diagnoses by clinicians among travelers returning from Africa.

## Methods

The GeoSentinel Surveillance Network (www.istm.org/geosentinel/main.html) is an international network of specialized travel and tropical medicine providers housed in sites in 23 countries on 5 continents, established through the International Society of Travel Medicine (www.istm.org/) and the Centers for Disease Control and Prevention (www.cdc.gov). Details of patient recruitment, structure, and function of GeoSentinel sites that systematically report on all ill travelers have been described (*2*). Data for ill travelers are collected during or after travel. Demographics, travel history, reason for travel, clinical symptoms, and diagnostic information are recorded anonymously on a questionnaire, and GeoSentinel sites enter the information into a central database. The best available reference diagnostic tests are used at each site to categorize illness into 1 of 23 syndromic groups and >500 individual diagnoses. Representatives from the sites enter each diagnosis as laboratory confirmed or probable; both are included in the analysis. The country or region of illness acquisition is identified on the basis of itinerary, known endemicity patterns, and incubation periods.

**Figure 1:**
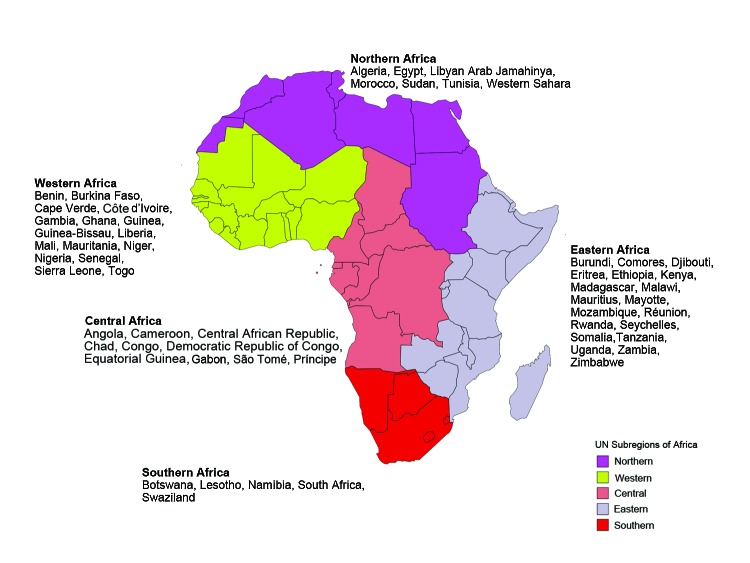
Regions of Africa as defined by the United Nations geoscheme (*5*). For persons whose country of exposure was unascertainable or missing but for whom all recent travel was to the same region of Africa, data were included in the final dataset.

**Figure 2:**
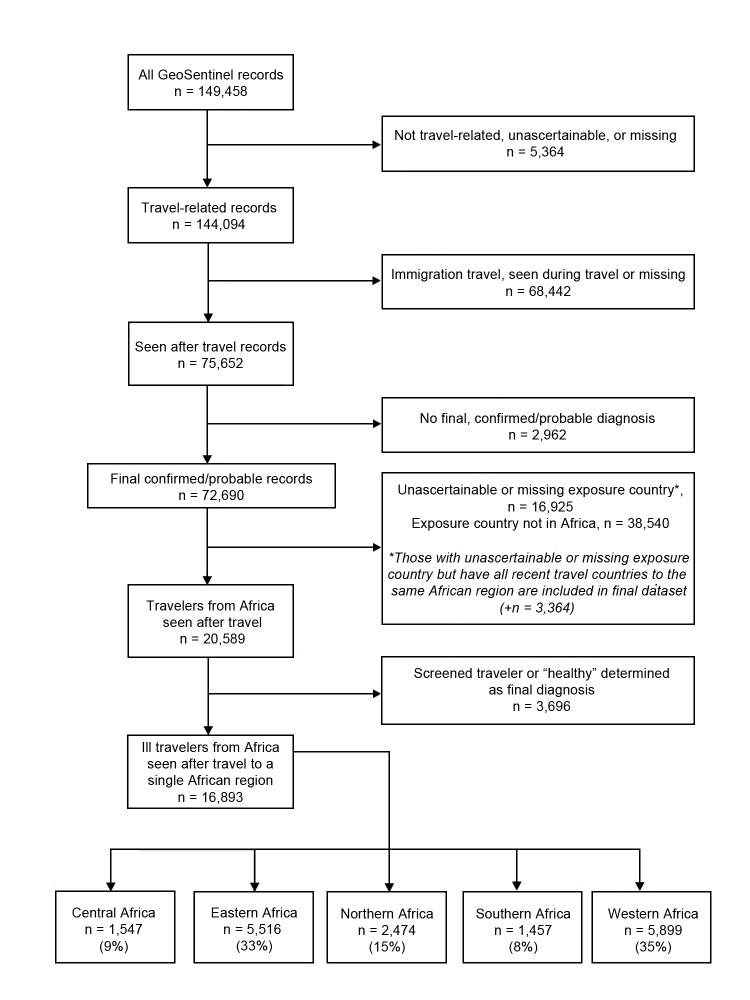
Flowchart for analysis of ill returned travelers from Africa reported in the GeoSentinel Surveillance Network, March 1997–May 2011. The United Nations geoscheme was used to classify Africa into subregions (*5*).

To study regional variation in the pattern of illnesses in persons who traveled to Africa, we used the United Nations geoscheme to classify Africa into subregions: Eastern, Central, Northern, Southern, and Western Africa (Figure 1) (*5*). An ill traveler returning from a country within an African region was considered to have been exposed to the causative pathogen in that region if the GeoSentinel database had a record of its occurrence there; or if the exposure was not defined in the record but the ill person traveled only to countries within that region. We included in our study all ill travelers listed in the GeoSentinel database during March 14, 1997–May 31, 2011 (Figure 2).

### Statistical Analysis

Demographic and travel characteristics of travelers to each African region were described by using frequencies and proportions for categorical variables and median and range for continuous variables. Analysis of *Plasmodium falciparum* malaria trends during 2007–2011 was calculated on the basis of monthly counts of ill returned travelers with febrile systemic illness that were aggregated over the study period. Of 54 sites in the GeoSentinel database reporting during 2007–2011, a total of 35 sites reported consistently during this period. We used only data from those 35 sites for trend analysis. Data were analyzed by using SAS version 9.2 (http://support.sas.com/software/92/index.html).

## Results

We identified 16,893 ill travelers who returned from a single country or multiple countries within the same region in Africa during a 14-year period. Most acquired their illness either in Western (35%) or Eastern Africa (33%). Illness associated with travel to Southern Africa (8%) was least frequently reported (Figure 2).

### Patient Characteristics

Most ill travelers returning from all regions were 18–64 years of age (Table 1). The residence of travelers spanned 72 countries; travelers were most frequently from Germany (33%), United States (12%), Canada (11%), France (10%), and Switzerland (10%).

**Table 1:** Characteristics of ill travelers returning from Africa who were seen at GeoSentinel clinic sites, March 1997–May 2011*

Characteristic	No. (%) travelers by region

Total, N = 16,893	Central Africa, n = 1,547	Eastern Africa, n = 5,516	Northern Africa, n = 2,474	Southern Africa, n = 1,457	Western Africa, n = 5,899
Sex						
M	8,698 (52)	922 (60)	2,582 (47)	1,126 (46)	723 (50)	3,345 (57)
F	8,130 (48)	615 (40)	2,909 (53)	1,344 (54)	733 (50)	2,529 (43)
Age, y						
Median (range)	35 (0–92)	37 (0–92)	34 (0–89)	37 (0–87)	40 (0–87)	35 (0–84)
≤5	364 (2)	27 (2)	79 (1)	72 (3)	12 (1)	174 (3)
6–17	553 (3)	31 (2)	147 (3)	109 (4)	36 (2)	230 (4)
18–49	12,172 (72)	1,145 (75)	4,059 (74)	1,638 (67)	936 (64)	4,394 (75)
50–64	2,869 (17)	282 (18)	900 (16)	471 (19)	324 (22)	892 (15)
>65	853 (5)	52 (4)	304 (6)	170 (7)	147 (10)	180 (3)
Expatriate	1,066 (6)	276 (18)	315 (6)	54 (2)	22 (2)	399 (7)
Travel reason						
Business	2,710 (16)	490 (32)	797 (14)	211 (9)	187 (13)	1,025 (17)
Volunteer	2,438 (14)	310 (20)	975 (18)	139 (6)	77 (5)	937 (16)
Student	419 (2)	29 (2)	162 (3)	14 (1)	28 (2)	186 (3)
Tourism	7,914 (47)	254 (16)	2,932 (53)	1821 (74)	1128 (78)	1,779 (30)
VFR†	3,304 (20)	442 (29)	622 (11)	276 (11)	34 (2)	1,930 (33)
Hospitalized	2,718 (16)	319 (21)	628 (12)	264 (11)	71 (5)	1,436 (25)
Died	11 (<1)	3 (<1)	2 (<1)	0	0	6 (<1)
Sought pretravel advice‡						
Yes	8,752 (52)	775 (50)	3,387 (61)	860 (35)	875 (60)	2,855 (48)
No	4,721 (28)	383 (25)	1,172 (21)	1,038 (42)	345 (24)	1,783 (30)
Don’t know	3,420 (20)	389 (25)	957 (17)	576 (23)	237 (16)	1,261 (21)

*Values are no. (%) unless otherwise indicated. The United Nations geoscheme was used to classify Africa into subregions (*5*). †VFR, visiting friends or relatives. ‡Sought advice from clinician pertaining to recognition of illnesses in travel destination, preventive measures, and treatment.

Male travelers more commonly visited Central and Western Africa, and female travelers were more likely to visit Eastern and Northern Africa. Travelers visiting friends or relatives (VFR) more commonly visited Central (29%) and Western (33%) Africa than the other regions (2%–11%). Business travelers more frequently traveled to Central Africa (32%) than to other regions (9%–16%). Approximately three quarters of travelers to Northern (74%) and Southern Africa (78%) were tourists. Travelers to Northern Africa were less likely to have had a pretravel medical consultation (35%) than were travelers to other regions (50%–61%). Posttravel hospitalization rates were higher in travelers to Central and Western Africa (21%–25%) than in travelers to Northern and Southern Africa (5%–11%). The most frequent diagnoses among hospitalized travelers were *P. falciparum* malaria (45%), *P*. *vivax* malaria (4%), and unspecified febrile illness (<3 weeks) (3%).

### Deaths among Travelers to Africa

Deaths of 11 travelers were recorded after travel to regions of sub-Saharan Africa (Table 2). Ten were of male sex; 10 were adults (median age 50 years). Severe *P. falciparum* malaria predominantly acquired in Western Africa was the cause of death for 9 of the 11 travelers. Two deaths occurred in expatriates, and 6 male business travelers died of *P*. *falciparum* malaria.

**Table 2:** Deaths of ill travelers returning from Africa who were seen at GeoSentinel clinic sites, March 1997–May 2011*

Patient no.	Age, y/sex	Diagnosis	Region	Exposure country	Travel reason	Expatriate
1	66/M	Malaria, *Plasmodium falciparum*. Severe and complicated, noncerebral	Western	Burkina Faso	Business	No
2	68/M	Malaria, *P. falciparum*. Severe and complicated, cerebral	Eastern	Kenya	Tourism	No
3	50/M	Malaria, *P. falciparum*. Severe and complicated, cerebral	Western	Ghana	Business	Yes
4	61/M	Malaria, *P. falciparum*. Severe and complicated, cerebral	Western	Sierra Leone	Business	No
5	4/M	Pneumonia, bacterial, lobar	Eastern	Tanzania,	Accompanying parent on business	No
6	48/M	Malaria, *P. falciparum*. Severe and complicated, cerebral	Western	Ghana	VFR	No
7	47/M	*Mycobacterium tuberculosis*, pulmonary and extrapulmonary	Western	Unknown	VFR	No
8	57/M	Malaria, *P. falciparum*. Severe and complicated, cerebral	Western	Liberia	Missionary/volunteer/ researcher/aid worker	No
9	30/F	Malaria, *P. falciparum*. Severe and complicated, cerebral	Central	Equatorial Guinea	Business	Yes
10	53/M	Malaria, *P. falciparum*. Severe and complicated, noncerebral	Central	Angola	Business	No
11	40/M	Malaria, *P. falciparum*. Severe and complicated, cerebral	Central	Angola	Business	No
*Three patients with malaria who died did not receive chemoprophylaxis, 1 received mefloquine, and data were missing for the remaining 5 travelers. The United Nations geoscheme was used to classify Africa into subregions (*5*). VFR, visiting friends or relatives.						

### Northern Africa

Travel to Northern Africa was predominantly characterized by acquisition of gastrointestinal illnesses, comprising 66% of the 16,893 travel-related illnesses from this region and 7 of the 10 most frequent diagnoses (Table 3). In contrast, gastrointestinal disorders from regions of sub-Saharan Africa represented 27%–40% of cases. There was no difference among regions for acute or chronic diarrhea, schistosomiasis, or other gastrointestinal disorders such as intestinal strongyloidiasis. Of the reported hepatitis A cases, 28 (47%) originated in Northern Africa. Analysis of the data for individual countries in the Northern Africa region did not show variation in type of diarrheal disease or gastrointestinal disease (data not shown).

**Table 3:** Diagnoses in descending order of frequency, by region of origin, for ill travelers returning from Africa who were seen at GeoSentinel clinic sites, March 1997–May 2011*

Total, N = 16,893	Illness and no. (%) travelers				
	Central Africa, n = 1,547	Eastern Africa, n = 5,516	Northern Africa, n = 2,474	Southern Africa, n = 1,457	Western Africa, n = 5,899
Malaria, *Plasmodium falciparum*. 2,118 (13)	Malaria, *P. falciparum*, 313 (20)	Viral syndrome, no rash, 444 (8)	Diarrhea, acute unspecified, 419 (17)	Rickettsia, tick-borne spotted fever, *R. africae, R. conorii, R. rickettsii* and other, 273 (19)	Malaria, *P. falciparum*, 1,484 (25)
Diarrhea, acute unspecified, 1,373 (8)	Viral syndrome, no rash, 114 (7)	Diarrhea, acute unspecified, 414 (8)	Diarrhea, chronic unknown, 262 (11)	Viral syndrome, no rash, 177 (12)	Viral syndrome, no rash, 396 (7)
Viral syndrome, no rash, 1200 (7)	Filaria, *Loa Loa*, 81 (5)	Malaria, *P. falciparum*, 291 (5)	Diarrhea, acute bacterial, 145 (6)	Diarrhea, acute unspecified, 95 (7)	Diarrhea, acute unspecified, 365 (6)
Diarrhea, chronic unknown, 791 (5)	Diarrhea, acute unspecified, 80 (5)	Diarrhea, chronic unknown, 254 (5)	Gastroenteritis, 107 (4)	Diarrhea, chronic unknown 69 (5)	*Giardia*, 209 (4)
Diarrhea, acute bacterial, 638 (4)	Diarrhea, acute bacterial, 48 (3)	Diarrhea, acute bacterial, 219 (4)	Rabies, postexposure prophylaxis, 107 (4)	Febrile illness, unspecified (<3 wk), 53 (4)	Diarrhea, acute bacterial, 207 (4)
Febrile illness, unspecified, <3 wk, 495 (3)	Febrile illness, unspecified, <3 wk, 46 (3)	Febrile illness, unspecified, <3 wk, 180 (3)	Irritable bowel syndrome, post infectious, 89 (4)	Respiratory tract infection (upper), 40 (3)	Febrile illness, unspecified, <3 wk, 184 (3)
*Giardia*, 491 (3)	Diarrhea, chronic unknown, 42 (3)	Respiratory tract inf (upper), 167 (3)	*Giardia*, 87 (4)	Bite or sting, insect, 33 (2)	Diarrhea, chronic unknown, 164 (3)
Respiratory tract infection (upper), 387 (2)	*Giardia*, 41 (3)	Bite or sting, insect, 142 (3)	Viral syndrome, no rash, 69 (3)	Rash, unknown etiology (non-febrile), 33 (2)	Respiratory tract infection (upper), 124 (2)
Irritable bowel syndrome, post infectious, 375 (2)	*Strongyloides*, simple intestinal, 34 (2)	Giardiasis, 133 (2)	Bite, dog, 52 (2)	Bite, insect; superinfected, 25, (2)	Irritable bowel syndrome, post-infectious, 110 (2)
Rickettsia, tick-borne spotted fever, 336 (2)	Malaria, severe and complicated, noncerebral, 27 (2)	Irritable bowel syndrome, post-infectious, 131 (2)	*Campylobacter*, 42 (2)	Bite, Tick 23 (2)	Malaria, species unknown 103 (2)

*The United Nations geoscheme was used to classify Africa into subregions (*5*).

Documentation of animal bites and the need for rabies postexposure prophylaxis (PEP) showed striking geographic variation. Of the 193 reports of bites on the continent, 23% were to travelers <18 years of age. Of the 184 who received rabies PEP, 21% were travelers <18 years of age. Travelers to Northern Africa accounted for 105 (54%) of the 193 bites from dogs, cats, and others (including monkey and human) reported in Africa (Table 4); in contrast, 16 (8%) bites were reported from Southern and Central Africa combined. Similarly, 107 (58%) of the 184 exposures requiring rabies PEP were reported from Northern Africa. Although Egypt was the most commonly visited country in Northern Africa (3 times the number of visits to Morocco), travelers to Morocco received the most bites (21 dog bites, 8 cat, 3 other).

**Table 4:** Nonmalarial illness among travelers returning from Africa who were seen at GeoSentinel clinic sites, March 1997–May 2011*

Illness/incident	No. travelers					
	Total	Region visited before illness				
		Central Africa	Eastern Africa	Northern Africa	Southern Africa	Western Africa
Parasitic infection by helminths						
Schistosomes	530	42	278	41	22	147
Unknown spp.	274	27	129	22	13	83
* Schistosoma mansoni*	147	9	89	14	5	30
* S. haematobium*	118	7	66	5	5	35
Filaria						
* Strongyloides*	195	34	78	13	6	64
Simple intestinal	191	34	78	12	6	61
Hyperinfection	4	0	0	1	0	3
Non-*Strongyloides*	140	102	6	1	1	30
* Loa loa*	86	82	1	0	0	3
* Onchocerca volvulus*	21	12	0	0	0	9
Other	31	9	4	0	1	17
* Wucheria bancrofti*	4	1	1	1	0	1
Vaccine-preventable disease	146	9	47	37	7	46
Hepatitis A	59	3	14	28	1	13
Influenza	24	0	11	4	2	7
Measles	5	0	0	1	3	1
Typhoid fever†	58	6	22	4	1	25
Bite wounds‡	193	5	47	105	11	25
Bite wounds necessitating rabies prophylaxis	184	4	38	107	13	22
Source of bite						
Dog	91	4	17	52	7	11
Cat	46	0	8	36	0	2
Other¶	56	1	22	17	4	12
Dengue (uncomplicated)	113	6	46	5	8	48
Tuberculosis	86	2	33	16	4	31
Pulmonary	43	2	14	14	2	11
Extrapulmonary	24	0	13	0	0	11
Miliary, disseminated	13	0	4	1	2	6
Meningitis	5	0	2	1	0	2
Multidrug resistant#	1	0	0	0	0	1
Acute HIV infection	44	4	21	0	5	14

*The United Nations geoscheme was used to classify Africa into subregions (*5*). †*Salmonella enterica* serotype Typhi. ‡Two travelers returning from Northern Africa and 2 returning from Southern Africa for whom bite wounds were not registered received rabies postexposure prophylaxis. ¶Other bites: monkey (23 bites); snake (3); rat (2); human, horse, puff adder, rabbit arthropoda, baboon, bat, hamster, leech, mangout, mouse, scolopendra, squirrel (1 each), missing data (14). #Resistance to rifampin and isoniazid.

### Regions within Sub-Saharan Africa

In contrast to the vast numbers of reports of gastrointestinal disease, febrile illnesses were uncommon in travelers to Northern Africa (4%). Conversely, 11%–47% of travelers returning from regions of sub-Saharan Africa had a febrile illness (Table 5). *P. falciparum* malaria was the most common cause of fever in returning travelers from sub-Saharan Africa, a finding consistent with those of previous studies (*6*,*7*). Of travelers who had malaria, 47% were VFR. Of travelers returning with malaria, 6% were <18 years of age. The proportion of febrile illness caused by malaria differed among regions: 2% of travelers returning from Southern Africa with febrile illness had malaria, compared with 69% and 67%, respectively, from Western and Central Africa. Conversely, African tick bite fever was the leading cause of illness in 273 (47%) of 579 travelers with fever returning from Southern Africa.

**Table 5:** Malaria in ill returned travelers from Africa seen at GeoSentinel clinic sites, March 1997–May 2011*

Illness	No. (%) travelers					
	Total, N = 16,893	Region visited before illness				
		Central Africa, n = 1,547	Eastern Africa, n = 5,516	Northern Africa, n = 2,474	Southern Africa, n = 1,457	Western Africa, n = 5,899
Febrile/systemic illness	5,505 (33)	626 (40)	1,474 (27)	219 (9)	579 (23)	2,607 (44)
Malaria†	2,789 (50.7)	416 (66.5)	515 (34.9)	37 (16.9)	12 (2.1)	1,809 (69.4)
*Plasmodium falciparum*‡	2,230 (40.5)	338 (54.0)	316 (21.4)	22 (10.0)	9 (1.6)	1,545 (59.3)
Uncomplicated	2,118 (38)	313 (50.0)	291 (19.7)	22 (10.0)	8 (1.4)	1,484 (56.9)
Severe noncerebral	104 (1.9)	27 (4.3)	23 (1.6)	1 (0.5)	1 (0.2)	52 (2.0)
Severe cerebral	61 (1.1)	13 (2.1)	12 (8)	0	1 (0.2)	35 (1.3)
* P. vivax*	197 (3.6)	19 (3.0)	122 (8.3)	9 (4.1)	0	47 (1.8)
* P.ovale*	138 (2.5)	21 (3.4)	26 (1.8)	2 (1)	0	89 (3.4)
* P.malariae*	84 (1.5)	20 (3.2)	17 (1.2)	2 (1)	0	45 (1.7)
Unknown species	179 (3.3)	26 (4.2)	44 (3.0)	3 (1.4)	3 (0.5)	103 (4.0)

*The United Nations geoscheme was used to classify Africa into subregions (*5*). †Sudan and Western Sahara are malaria-endemic countries. ‡Row totals indicate unique patients.

*P. falciparum* was the most common cause of malaria from all regions, including 100% of identified malaria cases from Southern Africa. *P. vivax* was proportionately more common from Eastern Africa (8%) than from other regions (0%–4%), and most of *P. ovale* and *P. malariae* cases were acquired in Central or Western Africa. Interregional seasonal variation in malaria acquisition was noted, although Western Africa was the only region that had a recognizable pattern of malaria cases, which the GeoSentinel sites reported more frequently from July through January.

A previous GeoSentinel study identified schistosomiasis as the most frequent helminthic infection reported in travelers returning from Africa, with regional variation noted (*8*). Seventy-three percent of reported strongyloidiasis cases were acquired in travelers returning from Eastern and Western Africa; 3% were reported in travelers to Southern Africa (Table 4). Acquisition of *Strongyloides stercoralis* from previous travel to, or birth in, other countries to which this infection is endemic cannot be discounted. Loasis accounted for 86 of 236 filarial infections, 82 (94%) of which were acquired from Central Africa, consistent with known endemicity; 50 (62%) of the 82 were from Cameroon, 12 from Gabon, 10 from Central African Republic, and 3 from Congo. Female travelers (58%) were more often infected by *Loa loa* than were men; volunteers (45%) and travelers VFR (30%) were the groups most often affected. Only 10% of cases were seen in tourists. Although largely an infection diagnosed in adults, 8% of cases were diagnosed in children (median age 10 years [interquartile range 7.0–16.5 years]).

Vaccine-preventable infections (VPI; i.e., hepatitis A, influenza, measles, and *Salmonella typhi* infection [typhoid]) taken together accounted for 0.9% of illnesses in travelers returning from Africa. The proportion of VPI from each region was comparable (Table 4). Among demographic groups, tourists were most likely to have VPI, most commonly hepatitis A and typhoid acquired in Northern Africa. Sex distribution was equal, and apart from 17 (29%) of the 59 hepatitis A cases that occurred in travelers <17 years of age, most VPI were diagnosed in adults. The acquisition of a VPI was not related to whether a pretravel consult had been sought.

During the 14-year study period, 86 cases of symptomatic tuberculosis were diagnosed in travelers returning from Africa, and an additional 159 travelers had positive tuberculin skin tests. Whether the positive skin tests were caused by acquisition of infection during travel or by prior exposure is unknown because no records of pretravel test results are available. Travelers returning from Central and Southern African regions, which are among countries with the highest rates of tuberculosis worldwide (*9*), accounted for 6 cases of symptomatic tuberculosis (Table 4).

Acute HIV infection was recorded for 44 persons. As was the case for tuberculosis, the number of acute HIV infections from Central and Southern Africa was lower than that from Eastern and Western Africa. No cases were diagnosed in travelers to Northern Africa.

Dengue is a common cause of illness in travelers to the Asia–Pacific region and Latin America (*2*); however, in our study, dengue acquired in Africa was diagnosed in as few as 113 travelers with febrile illness during the 14-year study period. Our data suggest that 81% of cases were acquired during travel to either Eastern or Western Africa (Table 4). Dengue was diagnosed equally in both sexes, and infection in travelers 18–49 years of age accounted for 81% of cases. Tourists were the major risk group for this illness.

## Discussion

Our study provides an evidence base of regional infectious disease exposures among travelers returning from Africa. These data show a profile of travel-related illness that differs with that of resident populations in these regions; this knowledge is essential in prioritizing preventive measures for the approximately 50 million travelers to Africa each year. Diarrheal and other gastrointestinal illnesses, hepatitis A, dog bites, and a very low proportion of febrile illnesses characterized the health of travelers returning from Northern Africa. In contrast, febrile illnesses were the predominant cause of clinic visits in travelers returning from sub-Saharan Africa, although considerable differences were evident in the etiology of fever in travelers from different regions. Malaria, which was most common in travelers returning from Central and Western Africa, was seen infrequently in travelers to Southern and Northern Africa. The incidence of helminthic infections also varied considerably: schistosomiasis and strongyloidiasis predominated in travelers returning from Eastern and Western Africa, but 82 of 86 *L. loa* infections reported in this study were diagnosed in travelers returning from Central Africa (Table 4). HIV infection and tuberculosis dominate the incidence of disease for much of sub-Saharan Africa and are an increasing concern for travelers, but our results show that these infections were rarely diagnosed in travelers at GeoSentinel Sites (Table 4).

Several factors likely contributed to our observations. Certain illnesses related to travel in Africa are more common in particular demographic groups. For instance, malaria is more common in travelers VFR than in tourists (*9*). In our study, malaria-related deaths occurred most often in men who traveled for business, a finding that may have implications for companies with expanding business interests in Africa. Pretravel medical advice and use of effective malaria prevention measures and chemoprophylaxis are essential for business travelers to areas of risk. Quantifying malaria risk is difficult, but the regional profiles presented here in which malaria predominated as a diagnosis are useful indicators (Tables 3, 5). High rates of malaria in Western and Central Africa reflect high malaria transmission levels in these areas. Entomologic inoculation rates (the number of infectious mosquito bites that a person receives in a certain time period) are a useful indicator of malaria risk. For example, the entomological inoculation rate for Bayma in Sierra Leone (Nov 1990–Oct 1991) was 884, compared with 1.5 for Kilifi Town in Kenya during the same period (*10*).

For some infections, such as the helminthic infections loaisis and onchocerciasis, vector distribution affects acquisition. Seasonal and climatic factors and degree of endemicity influence the likelihood of malaria and dengue transmission, whereas food and water hygiene, along with differences in sanitation, influence acquisition of diarrheal diseases. Illnesses that are commonly self-limiting and often have mild symptoms, such as influenza and dengue, are less likely to result in a visit to a clinic. Furthermore, travelers in a sub-Saharan Africa country who have fever may be examined by clinicians who will treat them empirically for malaria and thus misdiagnose another infection.

The finding that travelers were rarely seen in GeoSentinel site clinics for VPI, irrespective of the region visited, extends previous observations from Southern Africa (*4*) and remains a paradox, considering that rates of vaccination against these illnesses are historically <45% (*11*–*13*). We hypothesize that subclinical infection of children with hepatitis A or the low likelihood of adults or children with mild influenza-like illness to seek medical attention may account for low numbers of these 2 VPI reported from GeoSentinel sites. Most hepatitis A cases were in travelers to Northern Africa, which reflects the high rate of gastrointestinal infections from that region (Table 3). Our findings emphasize the importance of hepatitis A vaccination, which should be emphasized to health care providers and travelers alike, particularly when Northern Africa is the intended travel destination. 

The high number of animal bites and the subsequent need for rabies PEP in travelers returning from Northern Africa are likely to reflect bias because 97% of GeoSentinel-reported cases in travelers returning from Northern Africa who sought rabies PEP were reported to the Marseille, France, site, which is a reference center for management of suspected rabies exposures. However, the paucity of reported bites and need for rabies PEP at GeoSentinel sites among travelers returning from sub-Saharan Africa may in part reflect that travelers to sub-Saharan Africa were more often travelers VFR or long-term travelers, who may be more likely than tourists to Northern Africa to seek treatment at the time of exposure (*14*).

Sixty-seven percent of the world’s HIV-infected population resides in sub-Saharan Africa (*15*), and tuberculosis prevalence in some regions of Africa approaches 1% (*16*). Because 5%–50% of travelers report casual sexual experiences while traveling (*17*), it is surprising that so few cases of acute HIV infection were documented in travelers examined in GeoSentinel sites if risk behavior and endemicity of infection are high in many regions (*18*). Travelers who have symptoms of HIV and other sexually transmitted infections may seek care at specialty clinics rather than at GeoSentinel sites. However, because symptoms of acute HIV infection are commonly protean, and often manifest as a nonspecific febrile illness, travelers are as likely to go to GeoSentinel clinic sites as to specialist clinics. HIV should always be considered as a differential diagnosis in febrile returning travelers or in travelers who have clinical features compatible with HIV seroconversion illness. Regarding tuberculosis, although reactivation of latent infection may occur many years after acquisition, it is noteworthy that despite the large number of tuberculosis cases in most regions of sub-Saharan Africa, so few symptomatic cases occurred in travelers to Africa during the prolonged period of this study.

Previous studies of illnesses acquired by travelers to Africa have focused on illnesses acquired from sub-Saharan Africa as a whole (*2*,*19*), have been conducted at a single center (*20*,*21*), or have had analyses limited to a single disease or infection (*2*,*5*). Strengths of the current study are data capture from 54 international surveillance sites and >500 different diagnoses, enabling us to show regional patterns of illness in Africa. In addition, data were collected during an extended time period, which may offset acute spikes in reporting particular diagnoses that could skew the data.

## Conclusions

This study confirms that the likelihood of acquiring a specific infection or disease is not homogenous throughout the African continent. Medical practitioners examining returning febrile travelers from any region in sub-Saharan Africa must consider malaria as the first diagnosis. However, febrile patients, particularly those from Southern Africa, infrequently have malaria; other illnesses such as rickettsial infections are more common and should be strongly suspected. Concern about risk and possible damage to health is a determinant of whether a person will travel to a particular destination (*22*), so misconceptions should be corrected. However, because outbreaks reported, such as the recent reports of dengue virus in Angola (*23*) and Kenya (*24*) can rapidly alter exposure risk, up-to-date knowledge of patterns of disease is crucial.

During pretravel consultation, prevention of illness in travelers to different regions within Africa should be prioritized on the basis of the regional profile of diagnoses. For Northern Africa, advice should include detailed information about food hygiene and rabies prophylaxis. For countries in the Southern African region, malaria chemoprophylaxis and mosquito bite prevention measures should be used to prevent malaria acquisition during travel to regions in which malaria is endemic. The travel medicine advisor should be familiar with malaria-endemic and malaria-free areas in Africa to be able to provide suitable recommendations. Special attention should be paid to travelers VFR, who are most likely to acquire malaria, and to business travelers, whose malarial infection may be more likely to be fatal. Advice about preventing tick-borne rickettsial infections is essential for travelers to Southern Africa. Helminthic infections should be discussed. Preexposure and postexposure rabies vaccination, travelers’ diarrhea, and vaccine-preventable diseases should be discussed with travelers to destinations on the entire African continent.

Our analysis has some limitations: GeoSentinel Sites see a subset of all travelers to a particular country or region, and a main limitation of our study is an inability to calculate absolute risk for or true incidence of a disease from that country or region. Furthermore, not all returning travelers who are ill will seek care at a GeoSentinel site but instead will visit infectious diseases specialists, pediatricians, or nonspecialists. Children and adults with mild illness, such as an influenza-like illness, may not seek care at all. For 16,925 travelers in the GeoSentinel database, the name of the country in which they were exposed was missing or unascertainable. Of these, 3,364 persons had traveled to countries within the same African region and nowhere else and were therefore included in the dataset. The remaining 13,561 records were excluded from analysis. The effect of this removal of records on introducing bias is unknown. However, including them in the analysis would have risked including illnesses ascribed to 1 region but acquired in another. One GeoSentinel site contributed 25% of the final dataset, and these travelers were more likely to have traveled to Northern Africa (40%), be female (52%), and be a tourist (36%). Excluding this group did not substantially alter the profile of the final dataset. Northern Africa is viewed increasingly by European tourists as a Mediterranean-like destination in which risk for illness is lower than that in other parts of Africa, which may partially explain the low rate of pretravel consultation for travelers to Northern Africa.

The United Nations geoscheme for dividing Africa into 5 constituent regions is limited in that it was originally devised to enable statistical analysis of various parameters. Therefore, assignment of any 1 country into a particular area was for statistical convenience and was not meant to imply assumptions regarding health data. Thus, some countries, particularly those that border each other but are in different regions by the United Nations definition, may share health exposure risks for travelers, which therefore limits our study’s findings. However, because no division of Africa has been performed on health grounds, devising an arbitrary regional division would be equally as speculative and open to bias.

In conclusion, our study shows that health risks of travel to Africa are not uniform. Geographic profiling of illness acquired during travel to Africa facilitates targeted pretravel advice, expedites diagnosis in ill returnees, and may influence destination choices in tourism.
